# A cuproptosis-related lncRNA signature identified prognosis and tumour immune microenvironment in kidney renal clear cell carcinoma

**DOI:** 10.3389/fmolb.2022.974722

**Published:** 2022-09-14

**Authors:** Sheng Xin, Jiaquan Mao, Kai Cui, Qian Li, Liang Chen, Qinyu Li, Bocheng Tu, Xiaming Liu, Tao Wang, Shaogang Wang, Jihong Liu, Xiaodong Song, Wen Song

**Affiliations:** ^1^ Department of Urology, Tongji Hospital, Tongji Medical College, Huazhong University of Science & Technology, Wuhan, China; ^2^ Institute of Urology, Tongji Hospital, Tongji Medical College, Huazhong University of Science & Technology, Wuhan, China; ^3^ Department of Orthopedics, Tongji Hospital, Tongji Medical College, Huazhong University of Science & Technology, Wuhan, China

**Keywords:** kidney renal clear cell carcinoma, cuproptosis, lncRNAs, prognostic signature, tumor immune microenvironment

## Abstract

Kidney renal clear cell carcinoma (KIRC) is a heterogeneous malignant tumor with high incidence, metastasis, and mortality. The imbalance of copper homeostasis can produce cytotoxicity and cause cell damage. At the same time, copper can also induce tumor cell death and inhibit tumor transformation. The latest research found that this copper-induced cell death is different from the known cell death pathway, so it is defined as cuproptosis. We included 539 KIRC samples and 72 normal tissues from the Cancer Genome Atlas (TCGA) in our study. After identifying long non-coding RNAs (lncRNAs) significantly associated with cuproptosis, we clustered 526 KIRC samples based on the prognostic lncRNAs and obtained two different patterns (Cuproptosis.C1 and C2). C1 indicated an obviously worse prognostic outcome and possessed a higher immune score and immune cell infiltration level. Moreover, a prognosis signature (CRGscore) was constructed to effectively and accurately evaluate the overall survival (OS) of KIRC patients. There were significant differences in tumor immune microenvironment (TIME) and tumor mutation burden (TMB) between CRGscore-defined groups. CRGscore also has the potential to predict medicine efficacy.

## Introduction

Kidney cancer accounts for about 2% of newly diagnosed cancers and is the second most malignant tumor in the genitourinary system after prostate and bladder cancers (Siegel et al., 2021). Kidney cancer is characteristic of high incidence and mortality (Xu et al., 2019a; Xu et al., 2020a), among which the incidence in North America is much higher than in other countries and regions, reaching 14.9/per 100,000 (Padala et al., 2020). It was reported that about 180,000 people worldwide died of kidney cancer in 2020 (Sung et al., 2021). The subtype with the highest proportion is kidney renal clear cell carcinoma (KIRC), which has an awful degree of malignancy (Ravindranathan et al., 2021). Due to the asymptomatic characteristics of KIRC, about one-third of patients had distant metastasis when KIRC was initially diagnosed, and a high proportion of patients still had distant metastasis after operation (Padala et al., 2020; Ravindranathan et al., 2021). The specific median survival of patients with metastatic KIRC is only 1.2 years (Lohse et al., 2015). Due to the limited benefit of surgical treatment for patients with advanced metastatic KIRC, molecular targeted therapy, and novel immunotherapy have become the standard treatment for metastatic KIRC (Greef and Eisen, 2016). However, several clinical trials have shown that drug adjuvant therapy has limited clinical efficacy in patients (Meissner et al., 2018). Therefore, it is necessary to find effective indicators for drug selection and prognosis prediction to improve the survival prognosis of advanced metastatic KIRC patients.

Copper is an essential cofactor in the human body and actively maintains a very low level in cells through a cross-concentration gradient (Royer and Sharman, 2022). Copper is a double-edged sword. A higher concentration of copper will produce cytotoxicity and damage organs, such as liver injury and spleen injury (Yu et al., 2021; Guo et al., 2022). Copper can also induce tumor cell death and inhibit tumor transformation. Copper ionophore and copper chelator are considered anticancer agents (Hu et al., 2021a; Guo et al., 2021; Jiang et al., 2022). Initially, this copper-induced cell death was considered apoptosis (Bhatt et al., 2021; Chen et al., 2021). However, the latest research shows that copper ionophore induces a particular form of programmed cell death, which is called cuproptosis (Tsvetkov et al., 2022). It is found that the direct combination of copper and the fatty acylation components of the TCA cycle resulted in cuproptosis. A fatty acylation is a broad form of protein modification, which can change protein hydrophobicity and affinity for lipid bilayer (Resh, 2021). Protein acylation was also found to mediate necroptosis (Pradhan et al., 2021). Long noncoding RNA (lncRNA), an RNA transcript with a length of more than 200 nucleotides that does not encode protein (Bridges et al., 2021), in addition to mediating cancer-related biological processes, has a high predictive value for tumor diagnosis and prognosis (Peng et al., 2017; Huang, 2018; Xu et al., 2019b). At present, some studies have confirmed the relevance of cuproptosis-related genes with the prognosis and tumor microenvironment (TME) of KIRC by bioinformatics methods (Bian et al., 2022; Ji et al., 2022; Xu et al., 2022). Consequently, we used multi-omics bioinformatics analysis to elucidate the phenotypic characteristics of cuproptosis in KIRC and its correlation with the TME with the expression spectrum of cuproptosis-related lncRNAs.

Herein, a cuproptosis lncRNAs-related prognostic signature (CRGscore) was constructed to evaluate the immune characteristics and clinical prognosis of KIRC patients. Finally, it was confirmed that CRGscore was closely correlated with over survival (OS), clinicopathological characteristics, somatic mutation, tumor immune microenvironment (TIME), and medicine curative effects in KIRC patients, and had an accurate and stable ability to be the independent prognostic factor.

## Method

### Cell culture

The normal renal cell line HK-2, human renal cancer cell line 786-O and Caki-1 were all from the cell bank of the typical Culture Committee of the Chinese Academy of Sciences (Shanghai, China). RPMI 1640 medium, McCoy’s 5A medium, trypsin, streptomycin, and penicillin were purchased from Wuhan Boster Biological Technology, LTD. (Wuhan, China). Fetal bovine serum (FBS) was purchased from GIBCO (Grand Island, New York, United States). The medium of HK-2 and 786-O cell lines contained 90% RPMI 1640, 10% FBS, and 1% antibiotics (100 μ g/ml streptomycin and 100 U/ml penicillin). Caki-1 was cultured in 90% McCoy’s 5A supplemented with 10% FBS and 1% streptomycin and penicillin. All cell lines were cultured in 5% CO2 at 37°C. The culture medium was renewed every 2–3 days. Experiments were then performed on passaged three to five cells.

### Acquisition of cuproptosis-related LncRNAs

A total of 10 cuproptosis-related mRNAs (CRGs) were obtained from the research, including seven positive hits (FDX1, LIAS, LIPT1, DLD, DLAT, PDHA1, and PDHB) and three negative hits (MTF1, GLS, and CDKN2A) (Tsvetkov et al., 2022). 188 correlated lncRNAs were determined with |Cor| > 0.4 and *p*-value < 0.001. The “igraph” R package was employed to establish the co-expression network.

### Collection and processing of raw data

The transcriptional RNA-sequencing data of 539 KIRC tissue samples and 72 normal tissue samples were downloaded from the Cancer Genome Atlas (TCGA) database, and were also log2 transformed. After integrating clinical information, 526 KIRC samples with complete OS were included (Supplementary Table S1). The simple nucleoside variation data of 336 samples were also downloaded for somatic mutation analysis and tumor mutation burden (TMB) calculation. Then we applied the “caret” R package to categorize the 526 samples into the training cohort (*n* = 264) and test cohort (*n* = 262) (Supplementary Table S2).

### Identification of the prognostic-related differentially expressed LncRNAs

We calculated the cuproptosis-related lncRNAs expression profile in normal and tumor tissues, and identified 79 differentially expressed lncRNAs with |log2 (fold change)| >1 and false discovery rate (FDR) < 0.05 via the “limma” R package (Supplementary Table S3). Then we constructed further extracted the prognostic lncRNAs with the *p*-value<0.05 (Supplementary Table S4).

### Non-negative matrix factorization clustering

Prognostic lncRNAs were analyzed by using the non-negative matrix factorization (NMF) algorithm of the “NMF” R package, and the overall TCGA cohort was divided into clusters with distinct cuproptosis phenotypes (Devarajan, 2008). The optimal cluster number was selected by the cophenetic coefficient. The “prcomp” function in R was used for principal component analysis (PCA) to evaluate the distribution dispersion of clusters.

### Development and evaluation of the cuproptosis-related LncRNAs prognostic signature

After establishing the univariate COX regression model, we employed the LASSO regression analysis to avoid excessive overfitting and delete redundant lncRNAs, and obtained the prognostic model (CRGscore) containing four lncRNAs. The CRGscore formula is as follows:

CRGscore=Σ(exp⁡ Genei×coefficient Genei ) (1)

We calculated and obtained the CRGscore in the training set. In the Kaplan–Meier survival analysis via the “survival” R package, we defined the patients as high- or low-risk with the optimal cut-off value, which was obtained via the “surv_cutpoint” R function. The time-dependent receiver operating characteristic (ROC) curves were employed in the study with the “timeROC” R package. The true-positive rate and false-positive rate of ROC curves represent the percentage of patients who were correctly and incorrectly judged as dead according to the prognostic signature, respectively. The model was strongly verified in the test cohort and overall TCGA cohort.

### Clinicopathological correlation of cuproptosis-related LncRNAs signature

The CRGscore in clinicopathological subgroups of the TCGA-KIRC cohort was differentially analyzed. The prognostic value of CRGscore was verified by the univariate and multivariate COX regression analyses, and the clinicopathological parameters related to the prognosis were selected. A nomogram model contained CRGscore and selected parameters were established by the “rms” R package. The effectiveness of the nomogram model was evaluated with the calibration curves.

### Gene set enrichment analysis

Gene set enrichment analysis (GSEA) is an unsupervised algorithm to evaluate the biological signatures at the gene set level (Subramanian et al., 2005). We used the “clusterProfiler,” “enrichplot,” and “DOSE” R packages to perform GSEA on each sample in the cohort. Specific gene sets were supplied by the Molecular models database (MSigDB), including “c5. go.bp.v7.4. symbols”, “c2. cp.kegg.v7.4. symbols”, “h.all.v7.4. symbols”, “c2. cp.reactome.v7.4. symbols”, “c2. cp.biocarta.v7.4. symbols”, and “c2. cp.pid.v7.4. symbols”. Through the difference in GSEA score, we got the enrichment information of the risk score-defined groups in different biological processes.

### Analysis of the infiltration of tumor-infiltrating immune cells

ESTIMATE algorithm calculated the composition of TME of each sample, including stromal score, immune score, and tumor purity. ssGSEA algorithm obtained immune cell infiltration and the enrichment fraction of inflammatory responses of a single sample by analyzing the expression pattern of the marker gene set of specific tumor-infiltrating immune cells (TIICs) (Bindea et al., 2013; Charoentong et al., 2017).

### Evaluation of the medicine response

The Genomics of Drug Sensitivity in Cancer (GDSC) provided the prediction of chemotherapy and targeted drug efficacy, which was quantified by half inhibitory concentration (IC50) (Geeleher et al., 2014). “pRRophetic” R package realized this analysis (Yang et al., 2013).

### RNA extraction and real-time quantitative polymerase chain reaction (RT-qPCR)

TRIzol reagent (Beyotime, Jiangsu, China) was used to extract total RNA from cells. Total RNA was reverse transcribed into cDNA using the Servicebio®RT First Strand cDNA Synthesis Kit (Servicebio, Wuhan, China), and SYBR Green qPCR Master Mix (Servicebio, Wuhan, China) was used to perform RT-qPCR assay in ABI prism 7300 system (Thermo Fisher Scientific). GAPDH was used as a reference gene, and 2 − ΔΔ CT was used to calculate the fold change of target genes. The primers applied were displayed in (Supplementary Table S5). One-way ANOVA was used to analyze the significance of statistical results.

### Statistical analysis

R software (version 4.1.2) realized all the above statistical analyses. We used the Chi-square test to determine the distribution of clinic characteristics in subgroups (McHugh, 2013). When comparing two or more groups of continuous variables, Wilcoxon rank-sum test and the Kruskal–Wallis test were applied respectively (Rosner et al., 2003; Guo et al., 2013). The correlation analysis between continuous variables was calculated the Pearson’s analysis (Pripp, 2018). The “maftools” R package visualized the mutation spectrum. The “pheatmap” R package was to realize the heatmap. The Sankey diagrams were produced through the “ggalluvial” R package. Statistics were significant when *p*-value < 0.05 (*p < 0.05; * *p < 0.01; * * *p < 0.001; ns = no significance).

## Result

**FIGURE 1:**
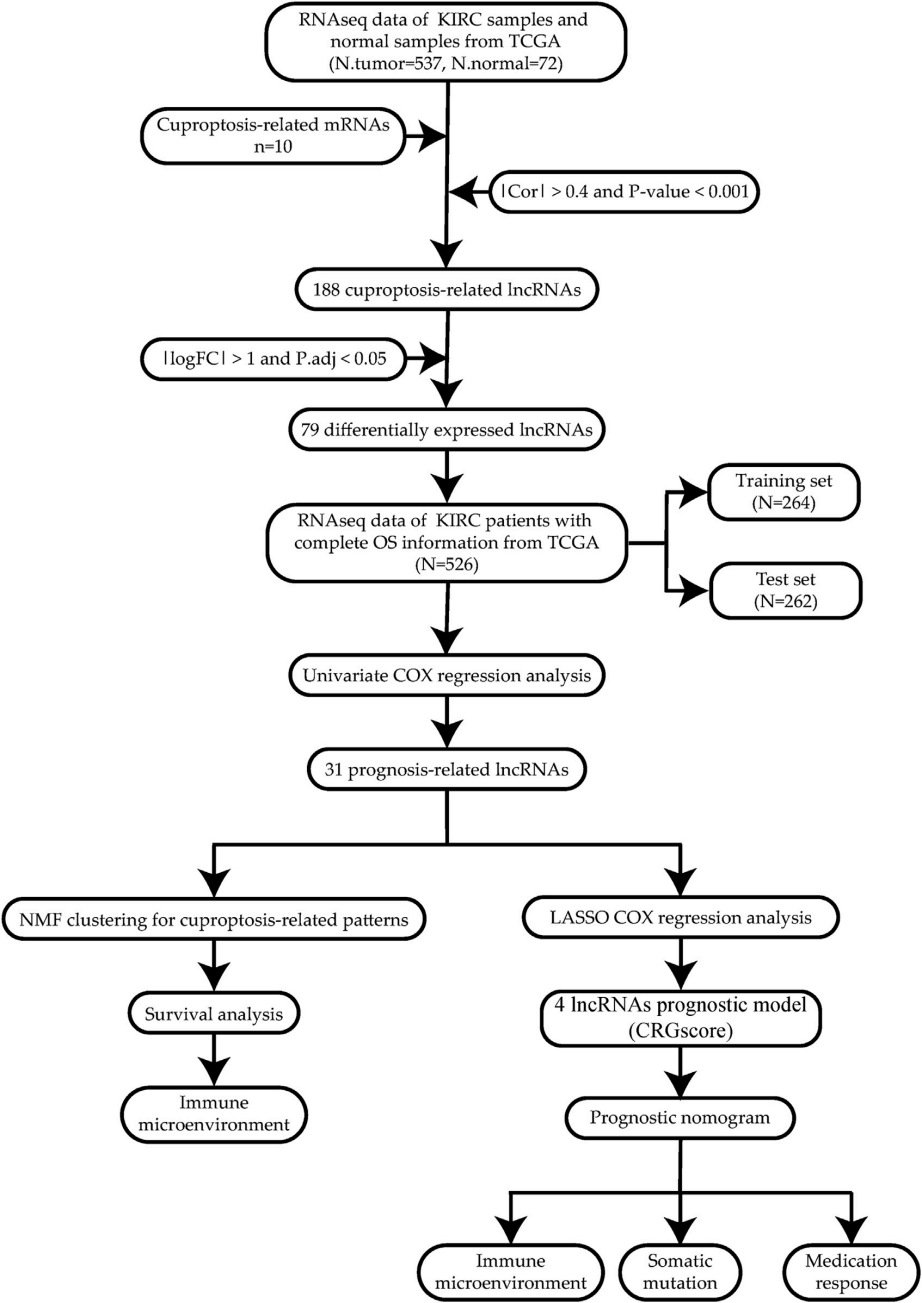
Research flow chart.

To reveal our research process concisely, Figure 1 is a flow chart.

### Acquisition of the cuproptosis-related lncRNAs

**FIGURE 2:**
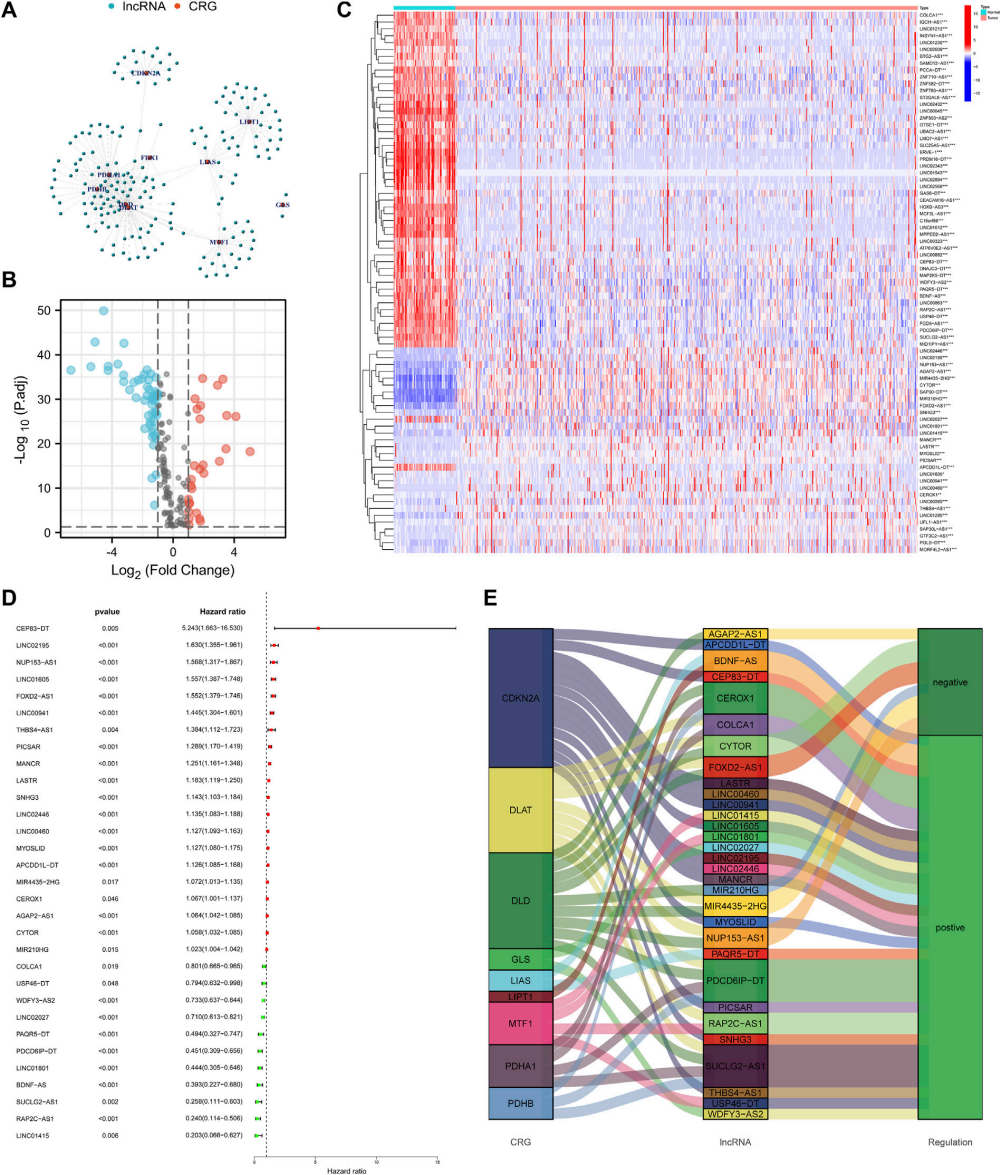
Selection of the cuproptosis-related prognostic lncRNAs differentially expressed in the KIRC. **(A)** Network of cuproptosis-related mRNAs and lncRNAs. **(B)** Volcano plot to identify differentially expressed lncRNAs. **(C)** Expression profile of the differentially expressed lncRNAs. **(D)** Univariate COX regression analysis to select prognostic lncRNAs. **(E)** Sankey diagram to visualize the relevance between mRNAs and lncRNAs.

Set the criterion with |Cor| > 0.4 and *p*-value < 0.001, 188 cuproptosis-related lncRNAs were identified (Figure 2A). Then we further selected 79 lncRNAs that were differentially expressed in the normal and KIRC tissues (Figure 2B). The expression profile of differentially expressed lncRNAs was shown in Figure 2C. Next step, we employed the univariate COX regression analysis and obtained 31 prognostic lncRNAs (Figure 2D). Figure 2E visualized the correspondence and regulation of the prognostic lncRNAs and cuproptosis-related mRNAs.

### Cuproptosis-related patterns in KIRC based on NMF clustering

**FIGURE 3:**
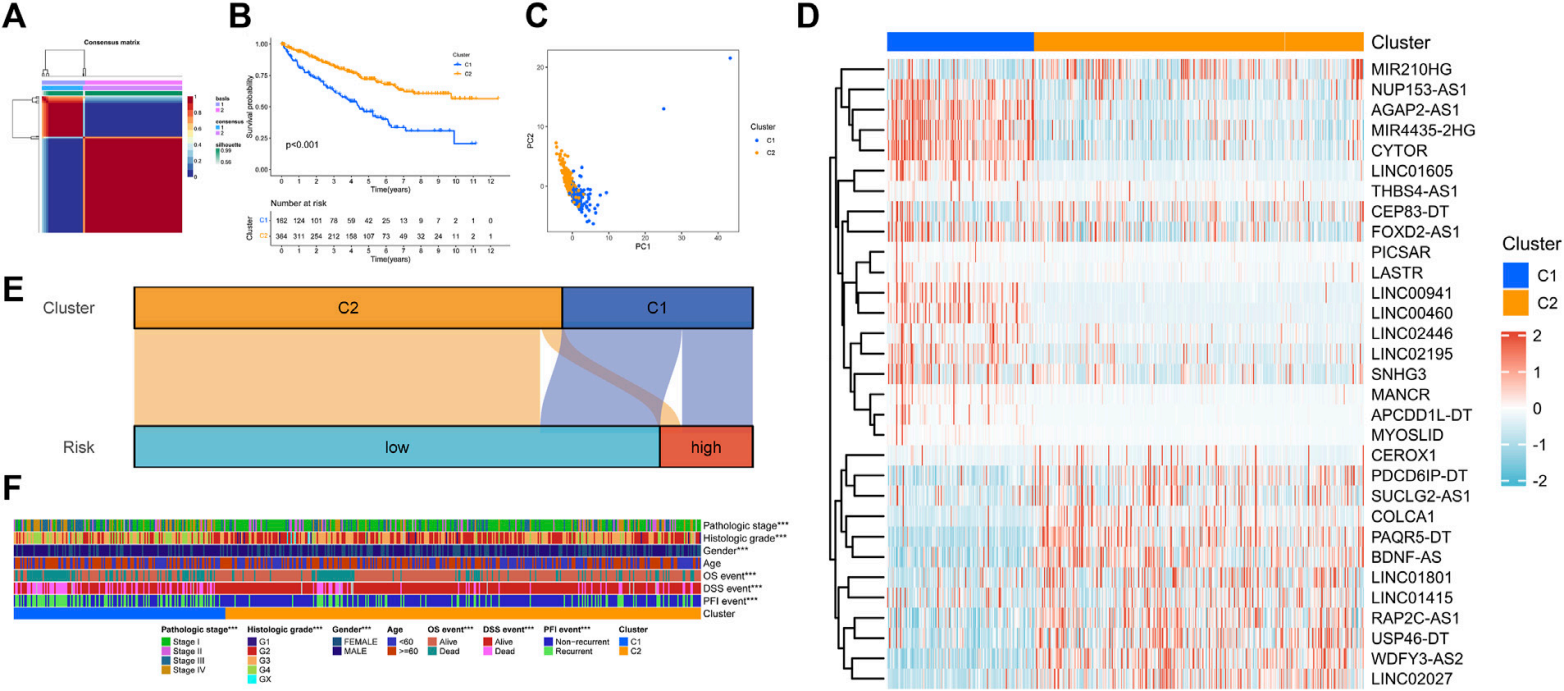
NMF clustering of cuproptosis phenotypes. **(A)** Consensus matrix heatmap. **(B)** Survival analysis of cuproptosis-related patterns. **(C)** PCA analysis. **(D)** Expression profile of prognosis-related lncRNAs. **(E)** Sankey diagram to reveal the distribution of samples. **(F)** Clinical relevance of cuproptosis-related patterns.

In clustering analysis, the optimal clustering number was determined as k = 2 (Figure 3A). Therefore, KIRC patients were divided into Cuproptosis. C1 and Cuproptosis.C2. In the Kaplan–Meier survival analysis, C1 had an obviously worse outcome (Figure 3B, *p* < 0.001). Then the direction of patient distribution of the two patterns was significantly discrete in the PCA analysis (Figure 3C). PC1 and PC2 reflected 24.7 and 14.5% characteristic differences in the expression profile, respectively. And the transcription profile heatmap visually shows the expression profile of the 31 prognostic lncRNA (Figure 3D). Figure 3E showed the attribution relationship of 526 samples in the CRGscore-defined groups and cuproptosis-related patterns. Additionally, we calculated the differences in clinicopathological features of patients in patterns and found the distribution of pathological stage, histological grade, gender, and prognostic events was distinct (Figure 3F).

### Tumor immune microenvironment in the cuproptosis-related patterns

**FIGURE 4:**
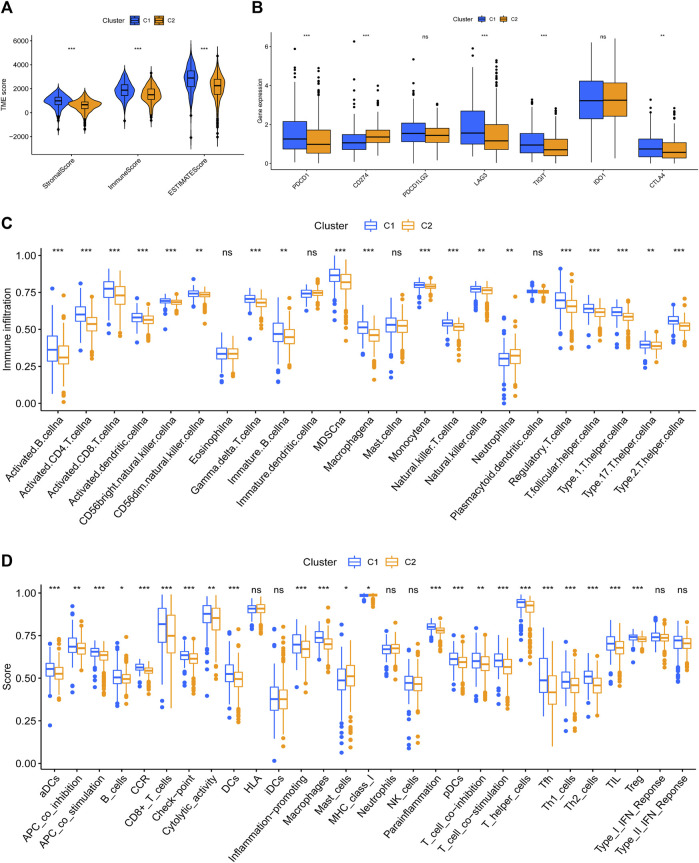
Tumor immune microenvironment between cuproptosis-related patterns. **(A)** The composition of TME in cuproptosis-related patterns. **(B)** Difference analysis of immune checkpoints expression. **(C,D)** TIICs-infiltrated phenotype **(C)** and enriched immune functions **(D)** of cuproptosis-related patterns.

It has been proved that the cytotoxicity caused by the imbalance of copper homeostasis leads to a variety of inflammation-related biological processes, such as apoptosis and oxidative stress (Chen et al., 2021). Quantitative analysis of ESTIMATE revealed obvious differences in the composition of TME between the two patterns, and C2 possessed the lowest immune score and highest tumor purity (Figure 4A). C1 had higher expression of PDCD1, LAG3, TIGIT, and CTLA4 (Figure 4B). And the infiltration level of most TIICs in C1 was significantly higher than in C2, except for eosinophils, DCs, mast cells, and neutrophils (Figure 4C). The results of the most immune function enrichment scores in C1 were also higher in C2 (Figure 4D).

### Establishing of CRGscore in the training cohort

We categorized the overall TCGA cohort into the training cohort (*n* = 264) and test cohort (*n* = 262), whose clinicopathological characteristics have no significant difference (Table 1).

**TABLE 1:** Clinical information of the training cohort and test cohort.

Characteristic	Training	Test	*p*-value	Method
*n*	264	262		
T stage, *n* (%)			0.736	Chisq.test
T1	131 (24.9%)	136 (25.9%)		
T2	37 (7%)	32 (6.1%)		
T3	89 (16.9%)	90 (17.1%)		
T4	7 (1.3%)	4 (0.8%)		
N stage, n (%)			0.857	Chisq.test
N0	121 (23%)	117 (22.2%)		
N1	7 (1.3%)	9 (1.7%)		
NX	136 (25.9%)	136 (25.9%)		
M stage, *n* (%)			0.628	Chisq.test
M0	213 (40.6%)	205 (39.1%)		
M1	37 (7.1%)	41 (7.8%)		
MX	12 (2.3%)	16 (3.1%)		
Pathologic stage, *n* (%)			0.825	Chisq.test
Stage I	130 (24.9%)	131 (25%)		
Stage II	32 (6.1%)	25 (4.8%)		
Stage III	61 (11.7%)	62 (11.9%)		
Stage IV	40 (7.6%)	42 (8%)		
Histologic grade, *n* (%)			0.623	Chisq.test
G1	4 (0.8%)	9 (1.7%)		
G2	118 (22.6%)	108 (20.7%)		
G3	102 (19.5%)	103 (19.7%)		
G4	36 (6.9%)	38 (7.3%)		
GX	2 (0.4%)	3 (0.6%)		
Gender, *n* (%)			0.667	Chisq.test
FEMALE	89 (16.9%)	94 (17.9%)		
MALE	175 (33.3%)	168 (31.9%)		
Age, median (IQR)	61 (52, 69)	60 (51.25, 69.75)	0.684	Wilcoxon

**FIGURE 5:**
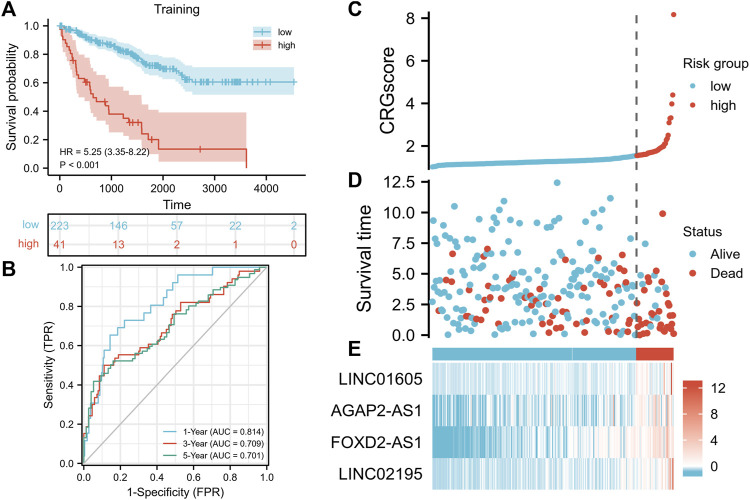
Construction of the prognostic signature. **(A)** Survival analysis of the CRGscore-defined training set. **(B)**Time-dependent ROC curves. **(C–E)** Distribution of the CRGscore **(C)**, OS outcomes **(D)**, and hub lncRNAs expression **(E)** in the training cohort.

We constructed a cuproptosis-related prognostic signature (CRGscore) to evaluate the predictive value of cuproptosis in KIRC. After constructing the univariate COX regression model, we selected the minimum standard coefficient according to the LASSO regression method. Finally, a total of four lncRNAs, and the risk score formula was: CRGscore = (0.113077 × LINC01605 expression) + (0.000545 × AGAP2-AS1 expression) + (0.130230 × FOXD2-AS1 expression) + (0.212716 × LINC02195 expression). We selected the optimal cut-off value (cut point = 1.547335) in Kaplan-Meier analysis according to the CRGscore and defined the patients as high-risk and low-risk. We drew the OS curve of the two groups of patients. And high-risk patients had obviously worse survival (Figure 5A, hazard ratio (HR) = 5.25 (3.35–8.22), *p* < 0.001). Then we evaluated CRGscore with the time-dependent ROC curve to reveal its accuracy. The AUCs were 0.814 at 1 year, 0.709 at 3 years, and 0.701 at 5 years (Figure 5B). And more deaths existed in high-risk patients (Figure 5C). The expression of LINC01605, AGAP2-AS1, FOXD2-AS1, and LINC02195 was up-regulated with the increase in CRGscore (Figure 5E).

### Verification of CRGscore in the test cohort and overall cohort

**FIGURE 6:**
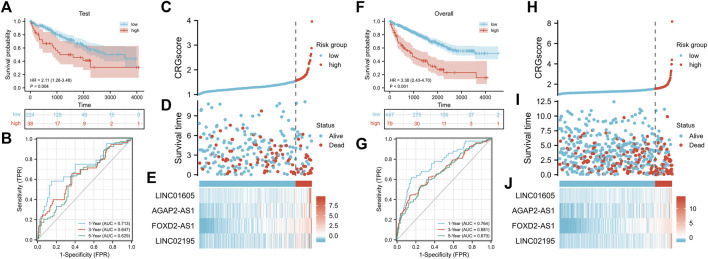
Verification of the prognostic signature. **(A)** Survival analysis of the CRGscore-defined test cohort. **(B)**Time-dependent ROC curves. **(C–E)** Distribution of the CRGscore **(C)**, OS outcomes **(D)**, and hub lncRNAs expression **(E)** in the test cohort. **(F)** Survival analysis of the CRGscore-defined overall cohort. **(G)**Time-dependent ROC curves. **(H–J)** Distribution of the CRGscore **(H)**, OS outcomes **(I)**, and hub lncRNAs expression **(J)** in the overall cohort.

To further verify the stability of CRGscore, the same CRGscore formula was applied to quantify the test set and overall cohort, and the same cut-off value was to divide samples. Consistent with the training set, high-risk patients possessed the worse prognosis event (Figure 6A, test cohort: HR = 2.11 (1.28–3.48), *p* = 0.004; Figure 6F, overall cohort: HR = 3.38 (2.43–4.70), *p* < 0.001). The AUCs were 0.713, 0.647, 0.629 in the test cohort (Figure 6B), and 0.764, 0.681, 0.670 in the overall cohort (Figure 6G). The distribution of CRGscore, OS status, and hub lncRNAs expression was also proved to be consistent (Figures 6C–E, and Figures 6H–J).

### Association of CRGscore with clinicopathologic features in KIRC

**FIGURE 7:**
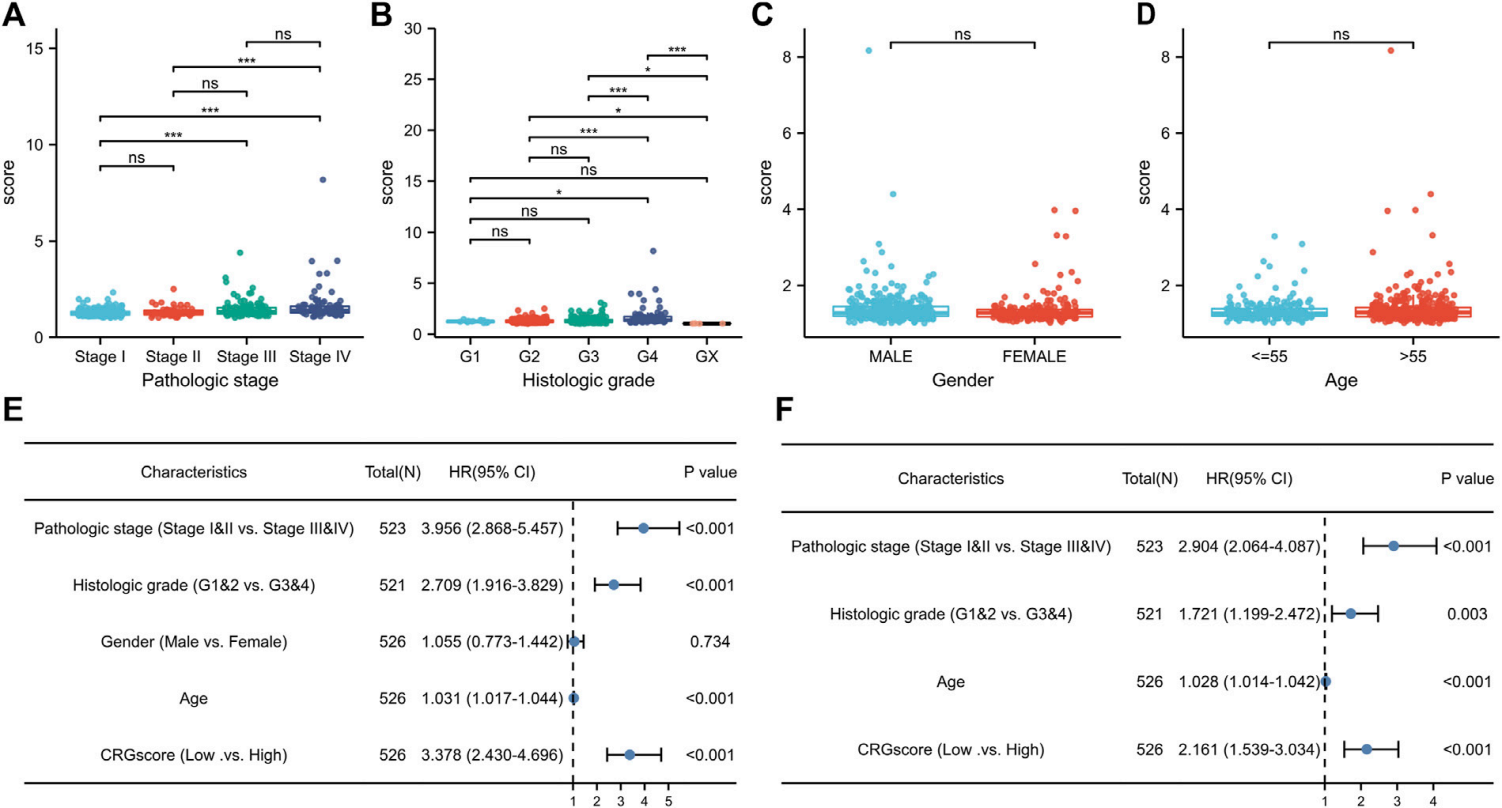
Clinical significance of the prognostic signature. **(A–D)** Clinic relevance of CRGscore, including pathologic stage **(A)**, histologic grade **(B)**, gender **(C)**, and age **(D)**. **(E,F)** Evaluation of the prognostic value with the univariate **(E)** and multivariate **(F)** Cox regression analyses.

Due to the clinicopathological heterogeneity of KIRC, we further investigated whether there was a significant relevance between CRGscore and clinicopathological features (Figures 7A–D). As shown in Figure 7A, CRGscore in stages III and IV was obviously higher than that in stages I and II. CRGscore was also significantly elevated in G4 (Figure 7B). CRGscore has no significant correlation with gender and age (Figures 7C,D). These results suggested that CRGscore could distinguish different clinicopathological features of KIRC patients. Furthermore, we found that pathological stage, histological grade, age, and CRGscore were independent prognostic factors, which had statistical significance in univariate and multivariate regression analyses (Figures 7E,F).

### 3.7 Construction of the nomogram model

**FIGURE 8:**
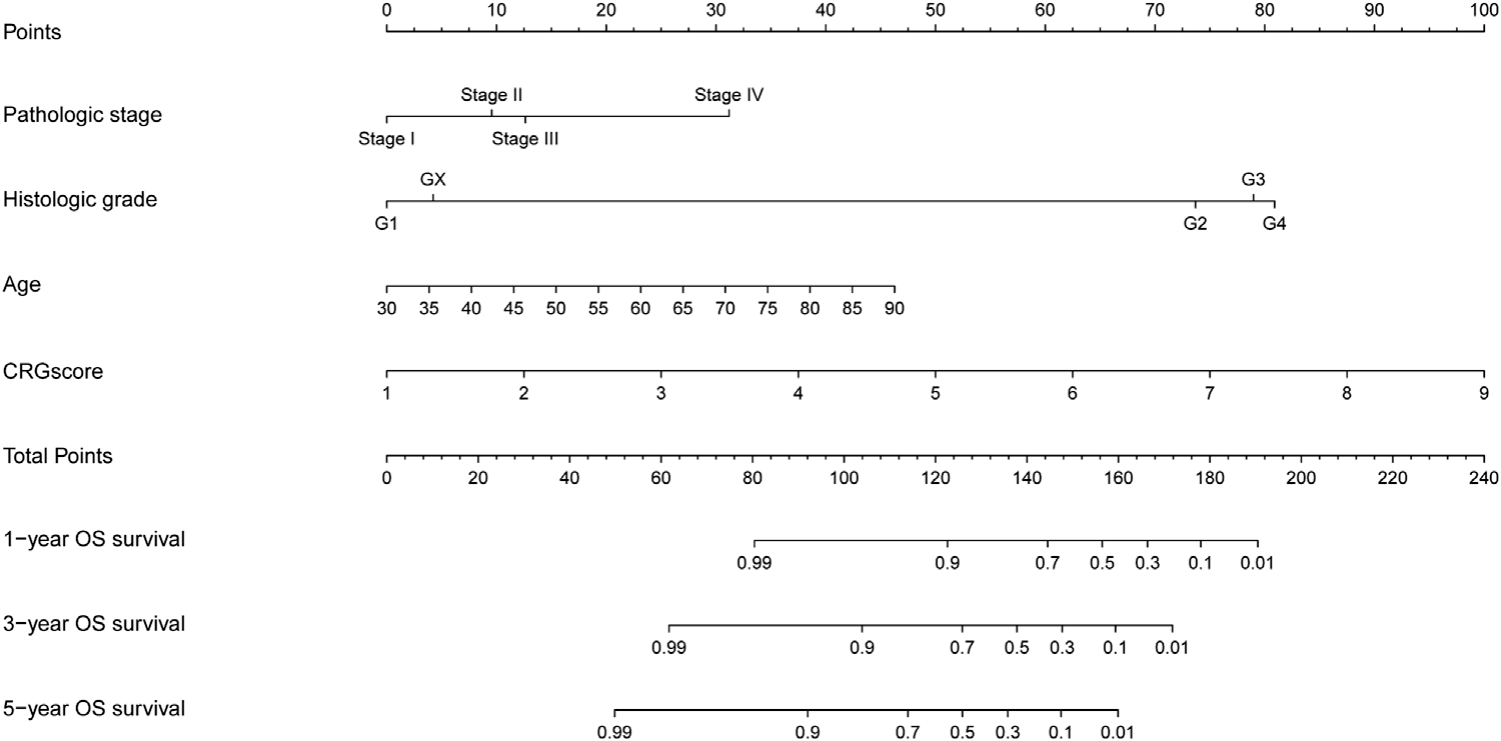
Nomogram to predict overall survival rate at 1, 3, and 5 years.

**FIGURE 9:**
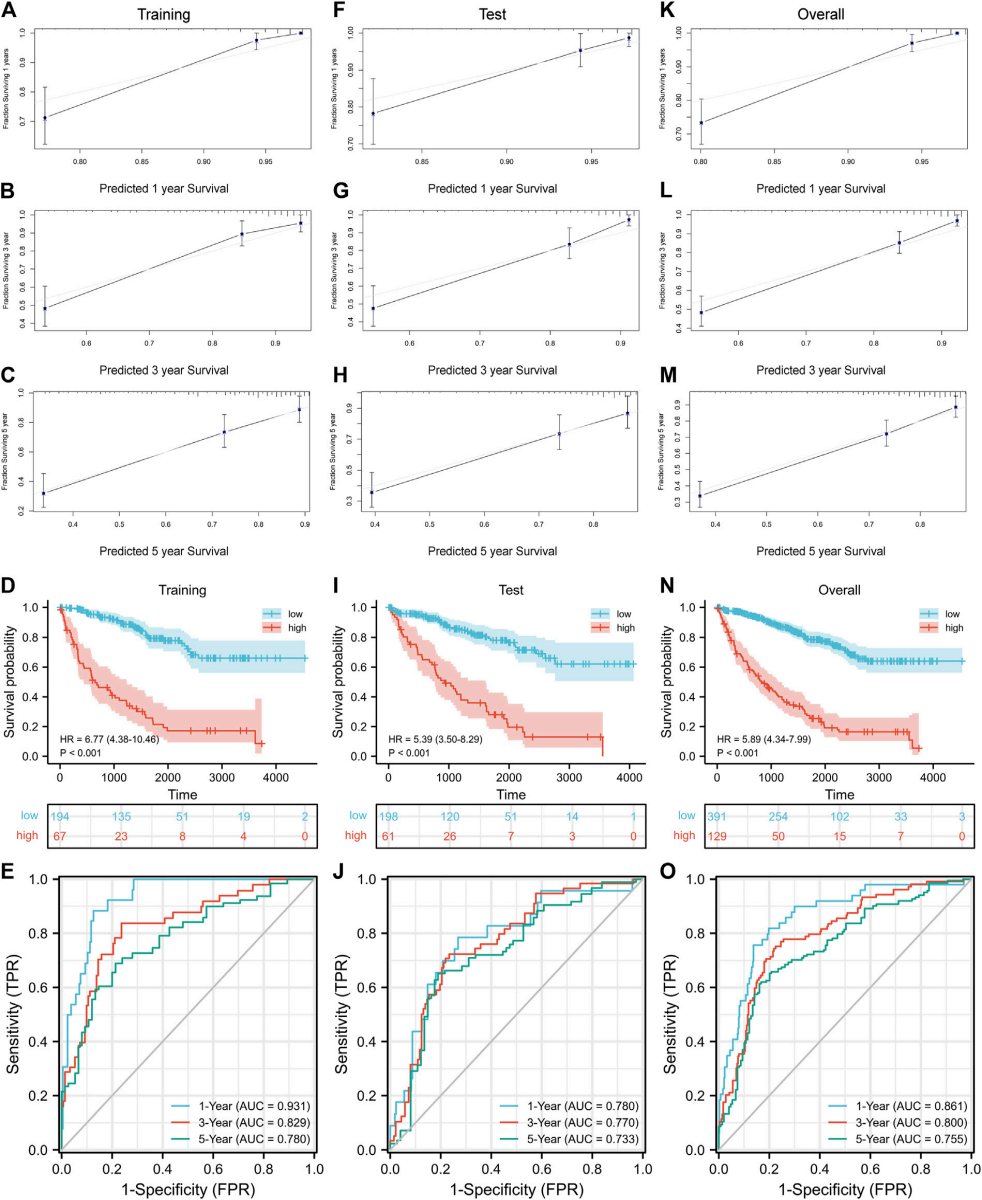
Evaluation of the nomogram. Confirmation of the nomogram coincidence at 1, 3, and 5 years with calibration curves in the training cohort **(A–C)**, test cohort **(F–H)**, and overall cohort **(K–M)**. Evaluation and verification of the prognostic value with survival analysis and ROC curves in the training cohort **(D,E)**, test cohort **(I,J)**, and overall cohort **(N,O)**.

Nomogram is a predictive model integrating multiple clinicopathological factors affecting prognosis. Four independent prognostic indicators were included in the nomogram model in this study, including pathological stage, histological grade, age, and CRGscore (Figure 8). Calibration curves were drawn in Figures 9A–C, F–G, K–M, which indicated the accurate coincidence of the nomogram in the training, test, and overall cohorts, respectively. After determining the optimal value (cut point = 0.764,068), the cohorts were divided. And the survival of the high-risk group was obviously worse (**d N**). The AUCs of the training cohort were 0.931, 0.829, and 0.780, the AUCs of the test coh Figures 9D,I, anort were 0.780, 0.770, and 0.733, and the AUCs of the overall cohort were 0.861, 0.800, and 0.755 (Figures 9E,J,O). The above results prove that the nomogram model provided a more accurate and stable tool for predicting prognosis.

### 3.8 Functional enrichment analysis

**FIGURE 10:**
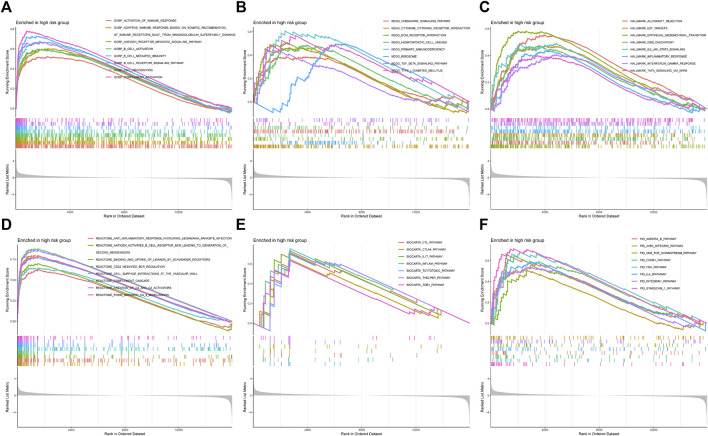
Functional enrichment analysis with GSEA. **(A)** Gene Ontology Biological Process (GOBP), **(B)** Kyoto Encyclopedia of Genes and Genomes (KEGG), **(C)** Hallmark, **(D)** Reactome, **(E)** BioCarta, and **(F)** PID.

To comprehend the potential biological function in high-risk patients, we characterized the expression data of the samples with GSEA (Figures 10A–F). Figure 10A showed that the high-risk group was highly concentrated in several immune-related biological processes, including activation of the immune response, adaptive immune response based on somatic recombination of immune receptors built from immunoglobulin superfamily domains, B cell activation, and so on. Some pathways, such as the chemokine signaling pathway and cytokine-cytokine receptor interaction, were also highly expressed (Figure 10B). And the high-risk group indicated several enriched hallmarks, such as allograft rejection, E2F target, G2M checkpoint, epithelial-mesenchymal transition (EMT), IL6-JAK-STAT3 signaling, and inflammatory response (Figure 10C). Additionally, some other immunological functions were also differentially expressed between the CRGscore-defined groups (Figure 10D).

### Association of CRGscore with tumor immune environment in KIRC

**FIGURE 11:**
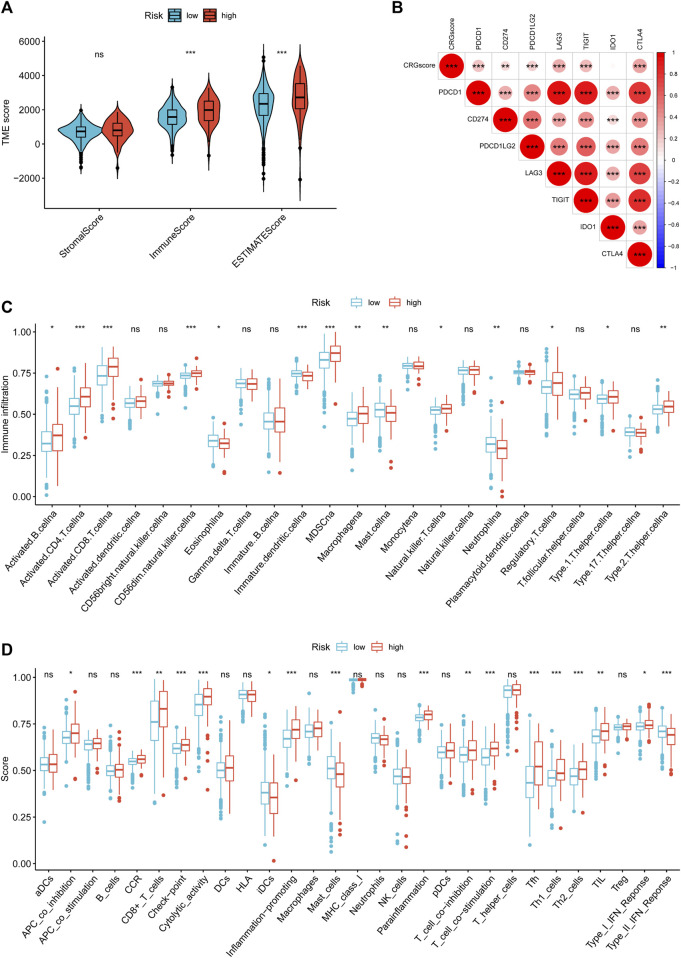
Immune cell-infiltrated phenotype of the CRGscore-defined groups. **(A)** The composition of TME was calculated through the ESTIMATE algorithm. **(B)** The correlation between CRGscore and the expression of immune checkpoints. **(C,D)** TIICs infiltration **(C)** and enriched immune functions **(D)** were calculated with ssGSEA.

To study the effect of cuproptosis on KIRC TIME, we employed ESTIMATE and ssGSEA to evaluate the infiltration level of TIICs in TME. The high-risk group with poor prognosis had higher immune and ESTIMATE scores, and lower tumor purity (Figure 11A). And CRGscore was significantly positively correlated with multiple immune checkpoints (Figure 11B). ssGSEA analysis demonstrated that various TIICs infiltrated significantly distinct, among which the infiltration of B cells, CD4 T cells, CD8 T cells, natural killer (NK) cells, Myeloid-derived suppressor cells (MDSC), macrophages, regulatory T (Treg) cells, Type 1 T helper (Th1) cells, and Th2 cells was obviously higher in the high-risk group. Contrarily, eosinophils, dendritic cells (DCs), mast cells, and neutrophils were more highly infiltrated in the low-risk group (Figure 11C). The enrichment scores of the immune function were shown in Figure 11D.

### Association of CRGscore with somatic mutation in KIRC

**FIGURE 12:**
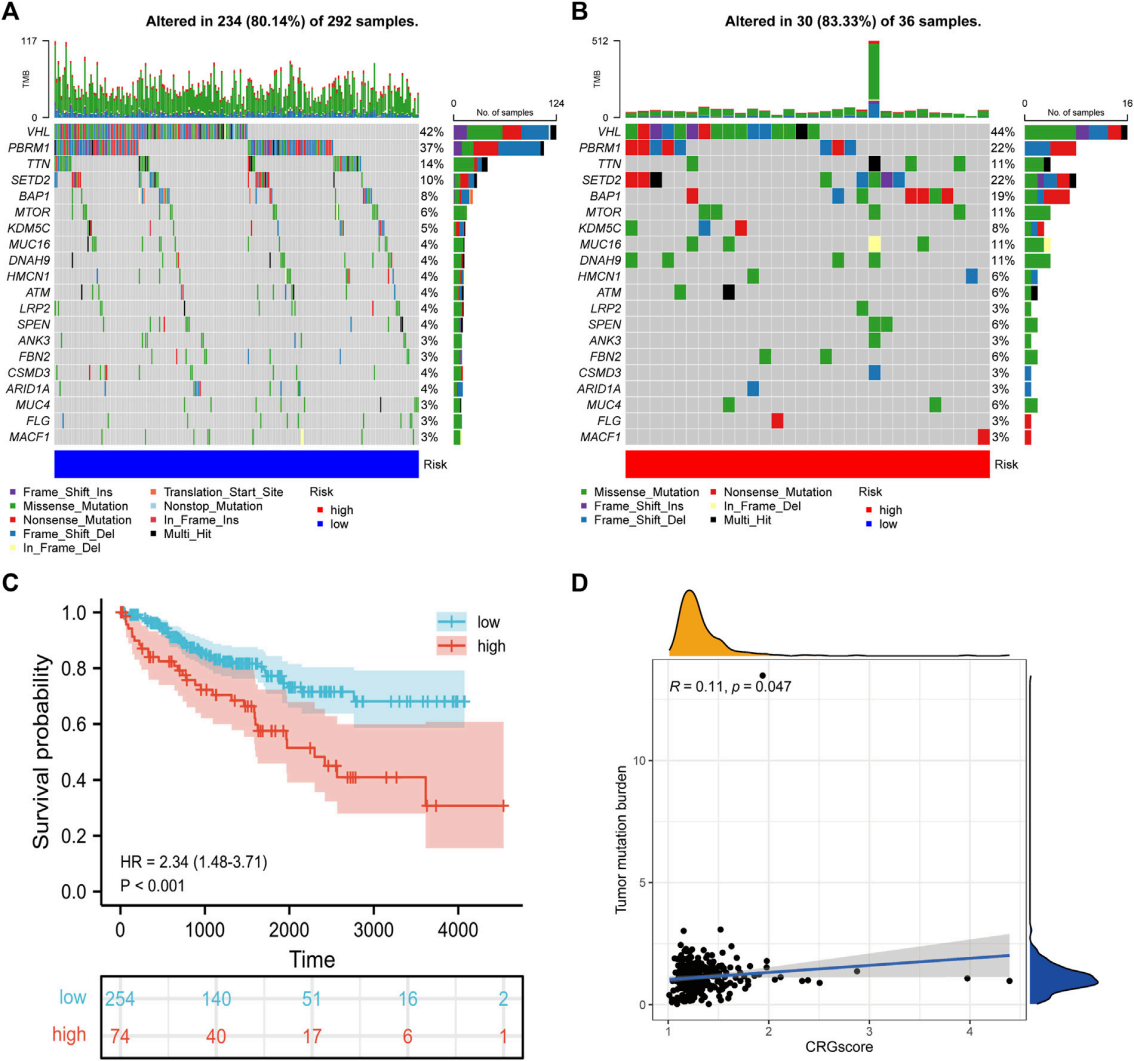
Somatic variants of the CRGscore-defined groups. **(A,B)** Mutation spectrum of the top 20 mutated genes. **(C)** Survival analysis of the TMB-defined groups. **(D)** The relevance of TMB with CRGscore was revealed through the correlation analysis.

Due to the damage to DNA caused by a high concentration of copper, we explored the correlation between CRGscore and somatic mutation of KIRC. As shown in the mutation spectrum in Figures 12A,B, the top three variated genes were VHL, PBRM1, and TTN in high-risk patients, while SETD2 was also highly mutated in low-risk patients. Survival analysis confirmed that high TMB trended to indicate a noticeably worse survival in KIRC (Figure 12C). CRGscore was also significantly positively correlated with the TMB in KIRC (Figure 12D).

### Association of CRGscore with response of drugs in KIRC

**FIGURE 13:**
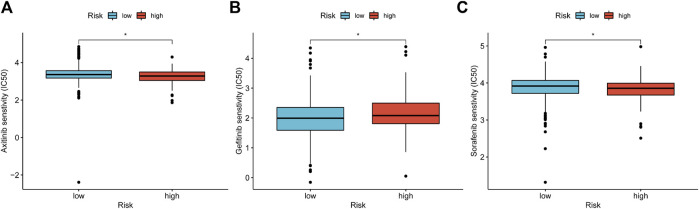
Predict potential drug treatment options. **(A–I)** The IC50 of chemotherapy and targeted drugs based on the TCIA database, including, Axitinib **(A)**, Gefitinib **(B)**, and Sorafenib **(C)**.

The first-line treatment for patients with advanced KIRC is chemotherapy and targeted therapy, thus we predicted the IC50 of different drugs in cancer cells. In Figures 13A–C, low-risk patients possessed significantly higher IC50 when treated with Axitinib and Sorafenib, which indicated these drugs possibly provide more benefits to high-risk patients. And Gefitinib is more suitable for low-risk patients.

### Verification with RT-qPCR

**FIGURE 14:**
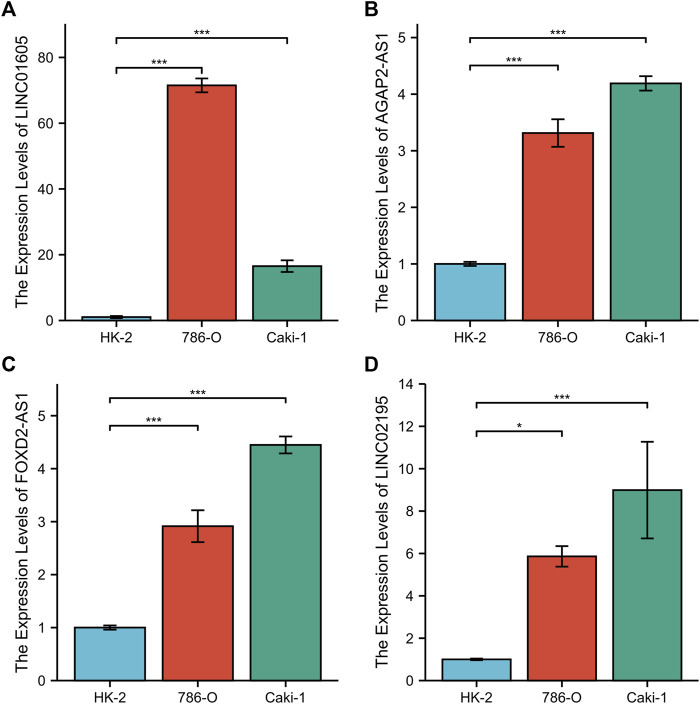
RT-qPCR of the four hub lncRNAs. **(A)** LINC01605, **(B)** AGAP2-AS1, **(C)** FOXD2-AS1, and **(D)** LINC02195.

To further validate CRGscore, we detected the expression of LINC01605, AGAP2-AS1, FOXD2-AS1, and LINC02195 in the normal renal cell line (HK-2) and human renal cancer cell lines (786-O and Caki-1) using RT-qPCR. The results showed that the mRNA expression of LINC01605, AGAP2-AS1, FOXD2-AS1, and LINC02195 in renal cancer cells was higher than that in normal renal cells (Figures 14A–D). This result confirms the reliability of our study.

## Discussion

KIRC originates from renal tubular epithelial cells and is a type of highly vascular heterogeneous malignant tumor (Hsieh et al., 2017; Atkins and Tannir, 2018). It is reported that more than 100,000 KIRC patients die each year due to tumor progression (Capitanio and Montorsi, 2016), so there are still limitations in assessing the survival of KIRC patients based on the current clinicopathological stages, and blood biochemical test results, and imaging evaluation (Hsieh et al., 2017). Therefore, finding new effective independent prognostic factors is the key to implementing individualized treatment and predicting the prognosis of the disease. Previous research has demonstrated that copper plays an anti-tumor role by regulating cell death, which is different from apoptosis, necroptosis, and some other known pathways. The study identified 10 cuproptosis-related genes through genome-wide functional deletion screening, including seven positive and three negative regulatory genes (Tsvetkov et al., 2022). The research on the biological function, treatment, and prognosis of cuproptosis in KIRC is blank. Therefore, in this study, we calculated and evaluated the cuproptosis-regulated lncRNAs with the phenotypic characteristics of multi-omics, to elucidate the potential regulatory mechanism and predictive value of cuproptosis in KIRC.

In this study, 526 valid samples from the TCGA-KIRC cohort were included and divided into the training set and test set. After identifying the prognostic lncRNAs differentially expressed in normal and KIRC tissues, we comprehensively employed the univariate and LASSO COX regression analysis to determine four lncRNAs (LINC01605, AGAP2-AS1, FOXD2-AS1, and LINC02195) and construct the prognostic signature (CRGscore) associated with the OS. LINC01605 has been demonstrated in triple negative breast cancer, nasopharyngeal carcinoma, colorectal cancer, and other cancers to promote tumor proliferation and invasion through multiple pathways (Hu et al., 2021b; Wang et al., 2022a; Zhao et al., 2022). AGAP2-AS1 was confirmed to be a prognostic marker of KIRC through the TCGA-KIRC cohort and a self-collected independent cohort (Gao et al., 2020; Nakken et al., 2021). FOXD2-AS1 can upregulate and activate the Notch signaling pathway in glioma, thus promoting tumor differentiation and proliferation (Wang et al., 2022b). LINC02195 was found to be a favorable prognostic marker in head and neck squamous cell carcinoma, which is opposite to the role in KRIC (Li et al., 2020). Therefore, the specific mechanism needs to be further explored. Then we assessed its prognostic value in the training set. CRGscore was confirmed as the independent prognostic factor of KIRC patients. Additionally, we integrated pathological stage, histological grade, age, and CRGscore to construct a more effective predictive tool for OS with the nomogram model.

In the GSEA, we found that several immune-activated and cell cycle-related biological processes were overexpressed in high-risk patients. The reversal of drug resistance of copper ions and copper compounds to cancer may involve the remodeling of the immune system (Valente et al., 2021). The molecular mechanisms of the cell damage caused by copper, such as oxidative stress, have a significant immune correlation (Khansari et al., 2009; Jomova and Valko, 2011; Chen et al., 2021). The E2F family plays a key role in the regulation of periods in cell division (Kent and Leone, 2019). High levels of E2F1 will lead to cell cycle arrest and apoptosis (Pützer and Engelmann, 2013). G2M checkpoint as a target can induce cell arrest and play an anti-tumor role (Reddy et al., 2019). EMT can enhance the migration and invasion of tumor cells, and endow cells with resistance to apoptosis. Moreover, EMT and inflammation have a mutually supportive relationship in tumors, which can enhance their malignant potential (Suarez-Carmona et al., 2017). IL6-JAK-STAT3 signaling pathway has a great impact on several ways of tumor progression, such as migration, invasion, and angiogenesis, and it also has the potential for prognostic evaluation in KIRC (Ni et al., 2020; Pan et al., 2020; Zhan et al., 2021).

Next, we analyzed the TIME of the CRGscore-defined group. The high-risk group was found to have an elevated immune score and a depressed tumor purity. Previous studies have revealed that patients with a high immune score or low tumor purity were trended to escape from tumor immunity and have a worse prognosis (Zeng et al., 2018; Gong et al., 2020). Highly infiltrated CD8 T cells and NK cells in high-risk patients have antitumor effects (Wu et al., 2020; Yanai et al., 2021). However, highly infiltrated MDSCs have the ability to limit the antitumor immunity and regulate TME (Weber et al., 2021). The regulatory mechanism of eosinophil infiltration in the TME of KIRC on tumor invasion and progression has not been clearly studied. However, Davis BP et al. believe that the infiltration of eosinophils in tumors is mostly related to the improvement of prognosis (Davis and Rothenberg, 2014). It is consistent with our results. And eosinophils respond to various stimuli, including the secretion of unique granule proteins that may kill tumor cells (Grisaru-Tal et al., 2020). Neutrophil to lymphocyte ratio has been proved to be an unfavorable prognostic factor for KIRC in several studies (Xu et al., 2020b; Życzkowski et al., 2020; Roussel et al., 2021), but we found that neutrophils infiltrate deeper in low-risk patients. Therefore, this requires further experimental validation and further exploration of the mechanism of neutrophils in the remodeling and regulation of TME. Moreover, high-risk patients had several highly expressed immune checkpoints, which were more likely to form the immunosuppressive microenvironment to lead to a worse prognosis (Gong et al., 2020). These results indicated that cuproptosis played an important regulatory role in TIME, which might affect the survival of KIRC patients. In the somatic variant analysis, VHL has the top mutation rate in KIRC, which directly leads to the imbalance of the hypoxia pathway (Schödel et al., 2016). In addition, SETD2, a key gene encoding modification-related enzyme, has a higher mutation rate in the high-risk group (Linehan and Rathmell, 2012). And we found that CRGscore was positively correlated with the TMB. Consistently, high TMB suggested a worse prognosis in KIRC patients. And studies have reported that a high level of immune checkpoint expression and TMB is an indicator of the response to immunotherapy (Chan et al., 2019; Sholl et al., 2020). Furthermore, CRGscore has the potential to guide the treatment strategy of drugs that have been used or have the potential to treat KIRC, including Axitinib (Rini et al., 2019) and Sorafenib (Rini et al., 2020), which are suitable for high-risk patients, while Gefitinib may be the ideal targeted medicine for low-risk patients (Li et al., 2021).

There are still some limitations of this study. As a retrospective study, our study was difficult to ensure the integrity and authenticity of patients’ clinical data, and the final study results are prone to bias. An independent cohort to validate our results is also valuable. The molecular mechanism of lncRNAs in KIRC still needs to be demonstrated *in vivo* or *in vitro*. Additionally, although CRGscore is related to the sensitivity of several anti-KIRC drugs, the mechanism of drug resistance in individual patients is complex, so this correlation needs further experimental or clinical verification.

## Conclusion

In conclusion, we first clustered the TCGA-KIRC dataset according to the lncRNA transcription level and divided it into two cuproptosis-related patterns with significant differences in prognosis and TIME. Then the cuproptosis-related signature (CRGscore) containing LINC01605, AGAP2-AS1, FOXD2-AS1, and LINC02195 was established and verified to quantify the cuproptosis phenotype and provide an effective and stable prognostic prediction tool for patients. And a comprehensive and systematic characterization of cuproptosis in terms of prognosis, TIME, somatic mutation, and drug sensitivity in KIRC was carried out.

## Data Availability

The original contributions presented in the study are included in the article/Supplementary Material, further inquiries can be directed to the corresponding authors.
